# Application of pH-Responsive Fucoidan/Chitosan Nanoparticles to Improve Oral Quercetin Delivery

**DOI:** 10.3390/molecules24020346

**Published:** 2019-01-18

**Authors:** Ana Isabel Barbosa, Sofia A. Costa Lima, Salette Reis

**Affiliations:** LAQV, REQUIMTE, Departamento de Ciências Químicas, Faculdade de Farmácia, Universidade do Porto, Portugal, Rua de Jorge Viterbo Ferreira, 228, 4050-313 Porto, Portugal; anabarbosa.cc90@gmail.com (A.I.B.); shreis@ff.up.pt (S.R.)

**Keywords:** antioxidant activity, in vitro release, marine polysaccharides, pH-sensitive nanoparticles

## Abstract

Polymeric nanoparticles based on fucoidan and chitosan were developed to deliver quercetin as a novel functional food. Through the polyelectrolyte self-assembly method, fucoidan/chitosan (F/C) nanoparticles were obtained with three different weight ratios (1/1, 3/1, and 5/1). The content of quercetin in the fucoidan/chitosan nanoparticles was in the range 110 ± 3 to 335 ± 4 mg·mL^−1^, with the increase of weight ratio of fucoidan to chitosan in the nanoparticle. Physicochemically stable nanoparticles were obtained with a particle size within the 300–400 nm range and surface potential higher than +30 mV for the 1F/1C ratio nanoparticle and around −30 mV for the 3F/1C and 5F/1C ratios nanoparticles. The 1F/1C ratio nanoparticle became larger and more unstable as the pH increased from 2.5 to 7.4, while the 3F/1C and 5F/1C nanoparticles retained their initial characteristics. This result indicates that the latter nanoparticles were stable along the gastrointestinal tract. The quercetin-loaded fucoidan/chitosan nanoparticles showed strong antioxidant activity and controlled release under simulated gastrointestinal environments (in particular for the 3F/1C and 5F/1C ratios), preventing quercetin degradation and increasing its oral bioavailability.

## 1. Introduction

Nature was always a rich source of compounds, usually associated with health-promoting properties and with therapeutic action in several pathologies [1]. One of the best known polyphenolic flavonols is quercetin, the most abundant antioxidant found in the human diet, sourced from a wide range of fruits and vegetables [2]. Quercetin also showed strong anti-inflammatory activity, as well as applications in the prevention of cancer and cardiovascular diseases [3,4]. The promising characteristics of this molecule caught the attention of several research groups, who exploit its benefits for medicine and nutrition purposes, ideally via oral administration of quercetin [5]. Yet, the stability and general characteristics of quercetin change according to food processing and pharmaceutical handling [6]. Variation in terms of pH, temperature, oxidation, and degradation can not only lead to a decrease in quercetin content, but also to the ineffectiveness of its promising therapeutic qualities [7]. Hence, the major drawbacks of quercetin are related to its low water solubility and, consequently, low oral bioavailability and instability in a physiological medium, as well as its high therapeutic dosage (about 500 mg twice a day) [8]. Since it is a lipophilic compound, the task of including quercetin in aqueous matrices is highly difficult, and the lack of stability throughout the gastrointestinal tract hampers its use in food applications. To elicit its health-promoting properties, quercetin is metabolized in the liver and absorbed in the intestine as a conjugate for distribution through the bloodstream [9]. Among other approaches, drug delivery systems were applied to enhance the oral absorption of quercetin and, hence, its oral bioavailability [8,10]. These can be defined as formulations or devices that enable the administration of therapeutic substances (both lipophilic and hydrophilic), improving efficacy and safety, by controlling the rate, time, and target-site of drug release [11,12]. So far, quercetin was merged with lipid-based delivery systems using nanoemulsion-based encapsulation [13,14,15,16,17], solid lipid nanoparticles [17,18,19,20,21], nanostructured lipid carriers [17,22,23,24,25], liposomes [26,27,28], glycerosomes [29], and other penetration enhancer-containing vesicles (PEVs) [30]. The main reported advantages of these systems include good stability and tolerability, and high encapsulation efficiency and targetability. In terms of limitations, the lipid-based systems present low stability in acidic pH (gastric sensibility) and a rapid release of the drug [5]. At first, the lipid-based systems seemed to be the most suitable choice for quercetin incorporation due to its lipophilic profile, but polymeric nanoparticles made of protein, synthetic, inorganic, and polysaccharide-based polymers are also very interesting [31,32,33]. Among the best features of quercetin-loaded polymeric nanoparticles, the possible use of natural ingredients, the prolonged drug release profile, and the production of small-sized nanoparticles stand out. As the major drawback, poor stability against aggregation is often mentioned. As an approach to overcoming the described limitations, some authors combined the best features of lipid systems with polymers to allow better stability and delivery of the compounds of interest. Some of the strategies involved the coating of liposomes with chitosan to achieve controlled and prolonged vesicle uptake via lung delivery [34] or to improve oral bioavailability and sustained release of hydrophilic drugs [35,36]. This approach was also explored to elicit an intestinal preferential release of quercetin, using a hybrid system composed of liposomes coated with cross-linked chitosan [37]. Considering the benefits of using quercetin as a nutraceutical and the convenience of oral administration, the use of natural polysaccharides with pH-responsive characteristics was the strategy used in this work to create nanoparticles that can resist harsh gastric conditions and provide enhanced quercetin intestinal absorption. 

The combination of two marine polysaccharides, fucoidan (a fucose-rich sulfated polysaccharide present in brown seaweeds) and chitosan (a biopolymer isolated from chitin), was explored for the design of nanoparticles able to resist the different gastrointestinal pH conditions [38,39]. Fucoidan is widely investigated because of its various biological properties such as anticoagulant, antiviral, antiangiogenic, anti-inflammatory, immunomodulating, and, most recently, antitumor activity [40]. Chitosan presents properties such as biocompatibility and biodegradability, and it can interact with polyanions, such as fucoidan, to form complexes [41]. The low cost, availability, and ease of chemical modifications make these marine biopolymers suitable for biomedical and pharmaceutical formulation [42].

To the best of our knowledge, there are no reports in the literature regarding the development of fucoidan/chitosan nanoparticles for the delivery of quercetin. Therefore, the purpose of this study was to develop quercetin-loaded polymeric nanoparticles that could have the potential to increase its bioavailability and therapeutic effects. The study explored three types of fucoidan/chitosan nanoparticles differing in their size and charge due to the different ratios between the two biopolymers. The physicochemical characterization of the nanoparticles included the pH-responsive profile determination, morphology analysis using transmission electron microscopy, and characterization in terms of size, zeta potential, polydispersity, and quercetin loading. Then, the stability of quercetin-loaded fucoidan/chitosan nanoparticles in biorelevant media and the antioxidant activity of quercetin were verified to validate the oral administration potential of the delivery system. 

## 2. Results and Discussion

In this study, fucoidan/chitosan (F/C) nanoparticles were obtained using a coacervation process [43]. The anionic fucoidan and the cationic chitosan were mixed at room temperature, and the interaction between both polyelectrolytes formed a complex with nanosized range as reported earlier [44,45]. The nanoparticles were produced applying three ratios between fucoidan and chitosan (1/1, 3/1, and 5/1), thus resulting in nanoparticles with different physicochemical properties. 

### 2.1. Fourier-Transform Infrared Spectroscopy Evaluation

**Figure 1:**
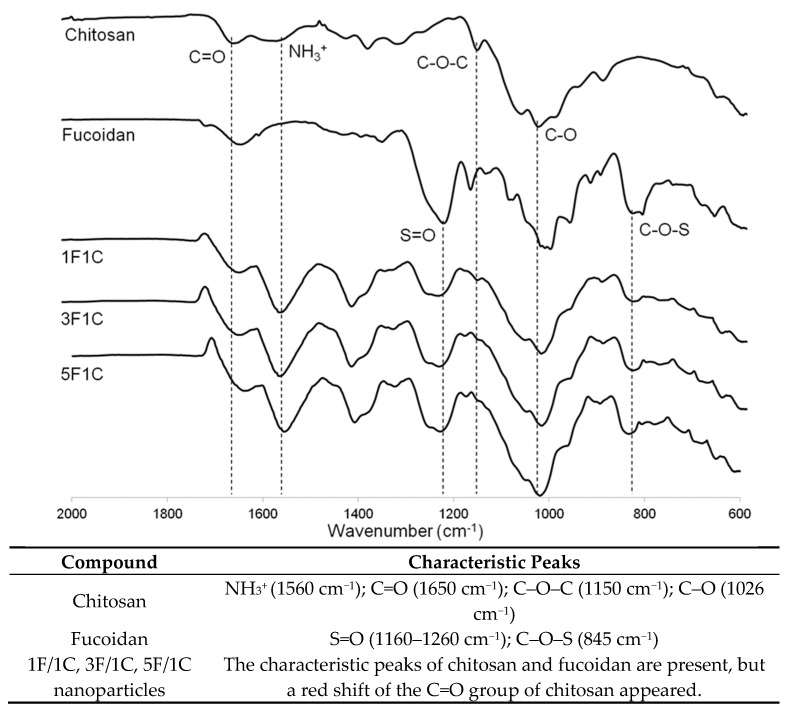
Fourier-transform infrared (FTIR) spectra of chitosan (C), fucoidan (F), and 1F/1C, 3F/1C, and 5F/1C nanoparticles, as well as a list of the characteristic peaks of each compound and produced nanoparticles.

In order to provide evidence on the interaction of the two marine polysaccharides upon nanoparticle production, infrared spectroscopy was performed. From the analysis of Figure 1, it is possible to monitor the presence of fucoidan and chitosan in the nanoparticles. The typical spectrum of fucoidan with S=O asymmetric stretching between 1160 and 1260 cm^−1^ and C–O–S stretching of the sulfate groups at 845 cm^−1^ can be observed in Figure 1 [46]. 

Furthermore, the characteristic peaks of chitosan are present at 1650, 1560, and 1150 cm^−1^, (N–H bending, C–N stretching from amide II, and asymmetric C–O–C stretching, respectively) and the C–O vibration at 1026 cm^−1^ is evidenced [47]. As expected, the fucoidan/chitosan nanoparticles showed the characteristic peaks of the fucoidan and chitosan spectra. However, a noticeable shift of the C=O group can be observed, which might be caused by a different environment around this group, as previously described [39,44,48,49]. Indeed, corresponding to a primary amide on the chitosan spectrum, the C=O stretching band appears around 1650 cm^−1^, and this is the reference wavenumber of chitosan alone. Once it is combined with other species, there is an addition of intermolecular interactions, causing a shift in the spectrum, possibly due to hydrogen-bonding and electrostatic interactions, explaining the changes in the environment of the described functional groups [50]. The FTIR analysis indicates that no covalent interactions were established between the two polysaccharides; thus, the nanoparticles were formed relying on the electrostatic interactions between the positively charged chitosan and the negatively charged fucoidan, in accordance with the production method [44].

### 2.2. pH-Responsive Profile of the Fucoidan/Chitosan Nanoparticles

The human organism is a complex environment, with different pH characteristics according to the physiology of each organ or tissue. In particular, the gastrointestinal tract exhibits a challenging range of pH values (pH 1–3 in the stomach, to pH 6–7.5 in the duodenum, jejunum, and ileum) [51], as well as several degradation and absorption processes. 

Among the many promising applications of marine polysaccharides, the possibility of achieving a nanoparticle able to resist the pH variations of the gastrointestinal tract was investigated in this work. The assessment was based on the properties of the fucoidan/chitosan nanoparticles in terms of size (to understand what happens to the structure) and the zeta potential (to understand what happens to the surface potential) (Table 1).

The 3F/1C and 5F/1C nanoparticles exhibited comparable particle sizes as the pH values increased from 2.5 to 7.4, suggesting that these nanoparticles remained stable and were insensitive to pH changes (Table 2). By contrast, the 1F/1C nanoparticles exhibited a significant response to pH variations. The 1F/1C nanoparticles swelled considerably as the pH level rose and decomposed at pH 6.0. The results can be explained based on the charge ratio of fucoidan to chitosan for each group. The charge ratios of fucoidan to chitosan for the 5F/1C and 3F/1C nanoparticles are 5:1 and 3:1, respectively. In the 1F/1C nanoparticles, both polysaccharides exhibited comparable charges. As the pH value was higher than 6.0, the deionized ammonium ions caused the nanoparticles to swell and disintegrate rapidly. As the pH value increased, the number of positive charges was insufficient to form stable nanoparticles, as already described in the literature [52]. In addition, the zeta potential of the nanoparticles is influenced by their composition (the weight ratio between chitosan to fucoidan) and the studied pH condition (Table 1). As the amount of fucoidan increased, the surface charge of the nanoparticles became negative. For 3F/1C and 5F/1C nanoparticles, the zeta potential values increased with the pH increase, while, for the 1F/1C nanoparticles, the opposite occurred. Likely, these effects are related to the functional groups of the nanoparticles (amino group on chitosan, and sulfate group on fucoidan) [38]. Therefore, the 1F/1C nanoparticles were more sensitive to pH changes than the 5F/1C and 3F/1C nanoparticles, with the latter determined as suitable for the intended oral administration.

### 2.3. Morphology Analysis

**Figure 2:**
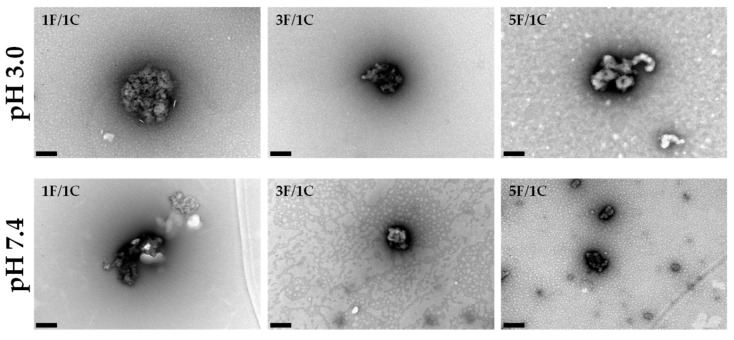
Fucoidan/chitosan nanoparticle morphology. Transmission electron microscopy (TEM) images obtained for the nanoparticles 1F/1C, 3F/1C, and 5F/1C. Scale bar = 200 nm.

The morphology of the nanoparticles was evaluated using transmission electron microscopy (TEM) as freshly prepared (pH 3.0) and after exposure to physiological pH values (Figure 2).

At pH 3.0, which was the pH of freshly prepared formulations, the 1F/1C nanoparticles were almost spherical and their size was around 400 nm. In the case of 3F/1C, the morphology was not regular and the size was around 300 nm. Looking to the 5F/1C ratio, the morphology resembled an aggregate of polysaccharides without a defined structure, with about 350 nm in the shortest diameter. These different morphologies were most certainly related to the ratio in which the nanoparticles were assembled. In the 1F/1C nanoparticles, the amount of oppositely charged groups in each polysaccharide is in the same ratio, allowing a more perfect interaction between each positive amino group interacting with a negatively charged sulfate group, performing a low-energy round shape. With the increasing concentration of fucoidan in the nanoparticle ratio, there are more negative charges to interact with positively charged chitosan, making it more difficult to rearrange the structures of each compound in the mixture. 

The fucoidan/chitosan nanoparticles were also observed at pH 7.4, to obtain information about the nanoparticles’ behavior under physiological conditions. In the obtained results, it is possible to visualize that the 1F/1C nanoparticles suffered disruption of their matrix, proving the sensitivity of this ratio under physiological conditions. In the other tested ratios, there was no major difference between pH 3.0 and 7.4. The nanoparticles looked the same size in both cases, revealing some stability. The 5F/1C ratio presented a more condensed polysaccharide structure, revealing morphological differences when compared with the initial pH conditions. The TEM images in Figure 2 identify the ratio 1F/1C as the most sensitive to environmental changes, in good agreement with the results detailed in Table 1.

### 2.4. Characterization of Fucoidan/Chitosan Nanoparticles Containing Quercetin

The size, size distribution, and surface properties of a nanoparticle are considered important features as they rule in vivo distribution, toxicity, and targeting potential. In addition, the physicochemical features determine drug loading, drug release, and nanoparticle stability [53]. For the evaluation of the three types of fucoidan/chitosan nanoparticles’ ability as an oral delivery system, quercetin was incorporated. Table 2 presents the physicochemical characterization of blank and quercetin (Q)-loaded fucoidan/chitosan nanoparticles, for the three ratios under study.

Particle size analysis revealed variability in the mean size (355–427 nm) for blank nanoparticles, and a narrow range of variability for drug-loaded nanoparticles (335–356 nm), which was expected considering the preparation method. The electrostatic interactions between fucoidan and chitosan produced smaller nanoparticles, and the incorporation of a bioactive molecule did not produce significant changes as the amount of fucoidan increased (3F/1C and 5F/1C nanoparticles). Similar observations were reported earlier in the literature [38]. The ratio 1:1 produced blank nanoparticles with a mean size around 430 nm. The addition of quercetin led to a significant reduction (*p* < 0.001) in mean size of the nanoparticles to 330 nm. The size distribution of the produced nanoparticles was below 0.3 for all formulations, which indicates a monomodal distribution suitable for oral administration [54]. The surface potential values obtained for fucoidan/chitosan nanoparticles were dependent on the ratio. The 1F/1C nanoparticles exhibited a highly positive zeta potential of +61 and +43 mV for blank and drug-loaded nanoparticles, respectively. The blank 3F/1C and 5F/1C nanoparticles were negatively charged (ca. −40 mV), and the incorporation of quercetin significantly (*p* < 0.01) reduced the surface charges toward less negative values (ca. −30 mV) (Table 1). The entrapment efficiency presented values ranging from 97% to 99%, revealing that most of the added drug was encapsulated in the polysaccharide matrix. These entrapment efficiency values resulted in high drug loading around 15% (*w*/*w*) in all nanoparticles, corresponding to a content of quercetin in the range 110 ± 3 to 335 ± 4 mg·mL^−1^, as the weight ratio of fucoidan to chitosan in the nanoparticle increased. Furthermore, the 3F/1C and 5F/1C nanoparticles showed a high absolute zeta potential charge; therefore, they were considered physically stable due to the electrostatic repulsions between nanoparticles [55].

### 2.5. In Vitro Release Assay of Quercetin

**Figure 3:**
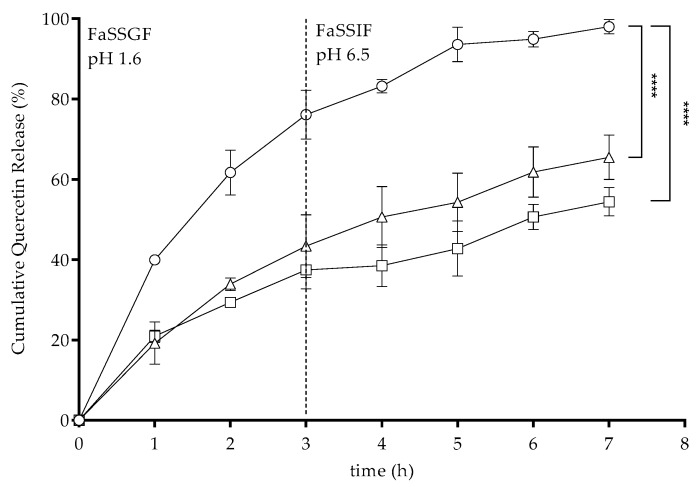
In vitro release of quercetin from loaded 1F/1C (circles), 3F/1C (triangles), and 5F/1C (squares) nanoparticles under simulated gastrointestinal conditions using fasted-state simulated gastric fluid (FaSSGF) and fasted-state simulated intestinal fluid (FaSSIF) at physiological temperature (37 °C). Data points correspond to means ± standard deviation for *n* = 3 replicates; **** p < 0.0001.

To evaluate the release of quercetin from the quercetin-loaded nanoparticles, in vitro release assays were performed using a dialysis cellulose diffusion technique. Considering the aim of this study to design fucoidan/chitosan nanoparticles for oral delivery, the quercetin release was evaluated under conditions that mimicked the gastrointestinal conditions. The use of biorelevant media was considered in order to provide a more realistic setting of the assay [56]. The composition of these media were close to the ones found in real organisms, making this a valuable tool to predict the performance of the drug in vivo [57]. The prepared commercial media were fasted-state simulated gastric fluid (FaSSGF; pH 1.6), followed by fasted-state simulated intestinal fluid (FaSSIF; pH 6.5). The nanoparticles were prepared as described, and the amount of quercetin released from each formulation was quantified using spectrophotometry. The obtained results are shown in Figure 3. 

For the 1F/1C ratio, the quercetin release reached about 80% after three hours under the gastric simulated environment, and total quercetin was released after two hours under intestinal conditions, confirming the pH-sensitive profile of this nanoparticles ratio, as detailed in Table 1 and Figure 2, where a complete destabilization of the nanoparticle was observed. For the 3F/1C and 5F/1C ratios, the release percentages were around 43% and 37%, respectively, in gastric media, while they were 65% and 54%, respectively, under intestinal conditions. No statistical differences were observed between the amount of quercetin released from 3F/1C and 5F/1C nanoparticles under both studied gastrointestinal conditions, corroborating the pH-resistant profile observed previously (Table 1). The obtained data revealed a possible interaction of FaSSGF with the nanoparticle structure, resulting in instability in the harsh gastric conditions, which could be related not only to the strong acidic pH, but also to the complex composition of this environment [58]. 

The quercetin release kinetics profiles from each polymeric nanoparticle were studied by analyzing the regression coefficients (*R^2^*) obtained after fitting into zero-order, first-order, Hixson–Crowell, Higuchi, and Korsmeyer–Peppas release kinetics models, as shown in Table 3. Considering the release kinetics profiles, the Korsmeyer–Peppas model best described the drug release mechanism in all ratios. Drug release is governed by a diffusion-controlled mechanism, described by the n-value. However, the n-value for quercetin release from 1F/1C nanoparticles was 0.4, while that for the 3F/1C and 5F/1C nanoparticles was 0.6 and 0.5, respectively. In the 1F/1C ratio, there was evidence of a Fickian diffusion of quercetin from the polysaccharide matrix. For the other two types of nanoparticles, a non-Fickian diffusion of the drug was observed, which is usually described as a combination of the effects of diffusion and erosion of the structure, providing a controlled rate release of the drug [59].

### 2.6. 2,2′-Azino-bis(3-ethylbenzothiazoline-6-sulfonic acid) Scavenging Activity Assay

**Figure 4:**
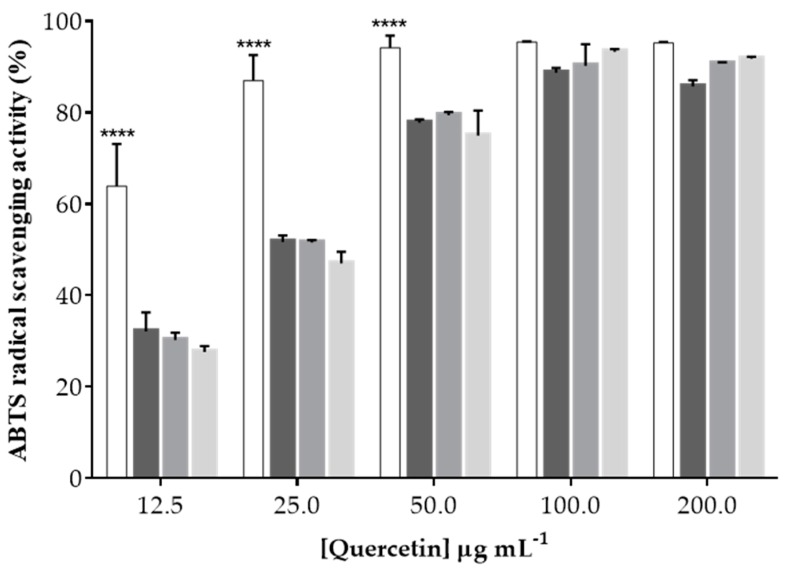
2,2′-Azino-bis(3-ethylbenzothiazoline-6-sulfonic acid) (ABTS) radical scavenging percentage of free quercetin (white bars) and quercetin-loaded nanoparticles (1F/1C, dark-gray bars; 3F/1C, gray bars; 5F/1C, light-gray bars). Data points correspond to means ± standard deviation for *n* = 3 replicates; **** p < 0.0001.

Given the antioxidant activity of quercetin, the 2,2′-azino-bis(3-ethylbenzothiazoline-6-sulfonic acid (ABTS) scavenging assay was performed to verify the ability of the nanoparticles to retain this biological effect, in addition to identifying a possible synergistic effect of the intrinsic antioxidant effect of fucoidan [60,61] (since it is one of the nanoparticles’ components) and quercetin. To fully understand the effect of an increasing amount of fucoidan in the formulation on the antioxidant activity, the same concentrations of polysaccharides in the nanoparticles were tested, although there were no statistically significant differences among the three ratios; the antioxidant effect for unloaded nanoparticles was around 10% ABTS radical scavenging activity (data not shown). However, when quercetin was added to the formulations, the antioxidant activity of the nanoparticles was similar to that of free quercetin for concentrations higher than 100 μg·mL^−1^, as shown in Figure 4.

Despite using the same concentration of quercetin in all nanoparticle combinations, the effect of quercetin was less visible at lower concentrations probably due to its entrapment in the nanoparticle matrix. Yet, it was possible to verify the presence of quercetin’s antioxidant activity when incorporated within these polymeric nanoparticles, confirming that one of the main interesting features of this nutraceutical was not compromised. 

## 3. Conclusions

In summary, fucoidan/chitosan nanoparticles were successfully formulated using the polyelectrolyte self-assembly method. Three fucoidan/chitosan ratios were used to produce nanoparticles, and it was shown that, with a higher ratio of fucoidan in relation to chitosan (3F/1C and 5F/1C), the nanoparticles were resistant to the gastrointestinal pH environments. It was found that the combination of fucoidan and chitosan as nanoparticles improved their physicochemical properties as oral delivery systems for quercetin, controlling its release under biorelevant simulated gastrointestinal environments, preserving its antioxidant activity through changing the fucoidan ratio. Therefore, the 3/1 and 5/1 fucoidan/chitosan ratios yielded nanoparticles with the potential to deliver bioactive polyphenols, which can be used as novel functional foods. 

## 4. Materials and Methods 

### 4.1. Materials

Fucoidan (from *Fucus vesiculosus*, molecular weight (MW) 50–190 kDa, pK_a_ 1.0–2.5), chitosan (MW 190–310 kDa, pK_a_ 6.5), quercetin, dimethyl sulfoxide (DMSO), and sodium chloride were supplied by Sigma-Aldrich (St Louis, MO, USA). Acetic glacial acid was obtained from VWR (Radnor, PA, USA). Double-deionized water was provided by a double-deionized water system (Arium Pro, Sartorius AG, Göttingen, Germany). The pH measurements were achieved using a Crison pH meter GLP 22 with a Crison 52-02 tip (Crison, Barcelona, Spain). 

### 4.2. Preparation of Fucoidan/Chitosan Nanoparticles

Fucoidan/chitosan nanoparticles were obtained with the polyelectrolyte self-assembly method using ultrasonication at room temperature according to a previously described method [39]. Briefly, the preparation process involved the mixing of the two polysaccharides under pulsed ultrasonication (pulse-on 3 s, pulse-off 7 s, totaling 30 s) using a probe sonicator (VCX130, Sonics and Material Vibra-Cell^TM^ with a CV-18 probe; 115 Newtown CT, USA) to promote the self-assembly of these compounds and produce the nanoparticles. Prior to this step, fucoidan was dissolved in double-deionized water and chitosan in 1% (*v*/*v*) acetic acid solution, using an ultrasound bath to enhance the solubility. With these solutions, different ratios of the fucoidan/chitosan nanoparticles were prepared, namely 1:1, 3:1, and 5:1. After the self-assembly, the formulations were filtered using an 800-nm Minisart^®^ Syringe Filter (Sartorius Stedim Biotech GmbH, Göttingen, Germany) for removal of large aggregates. To produce quercetin-loaded nanoparticles, the same procedure was performed adding quercetin in an equivalent 15% (*w*/*w*) of total F/C mass to the polysaccharide mixture before the sonication step, promoting its inclusion in the nanoparticle matrix. 

### 4.3. Characterization of Fucoidan/Chitosan Nanoparticles

#### 4.3.1. Average Size and Surface Potential Determination

The mean size, polydispersity index (PDI), and zeta potential of the formulations were determined using a ZetaPALS zeta potential analyzer (Brookhaven Instruments Corporation; Holtsville, NY, USA). In particle size measurements, six runs of 2 min were performed at room temperature for each assay. The zeta potential of the nanoformulations was determined using an electrode operating at a scattering angle of 90° at room temperature. For each assay, six runs of 10 cycles were performed.

#### 4.3.2. Morphology Assessment

The morphology of the nanoparticles was assessed by transmission electron microscopy (TEM). In each measurement, 20 μL of each formulation was placed in the copper grid for 1 min. The excess was removed, and then 0.75% (*w*/*v*) uranyl acetate was added to the sample as a contrast agent. The grid was exposed at the accelerating voltage of 60 kV. The images were obtained in a JEM-1400 transmission electron microscope (Jeol JEM-1400, JEOL, Ltd., Tokyo, Japan).

#### 4.3.3. Determination of the Entrapment Efficiency and Drug Loading

The amount of quercetin in each formulation was determined by centrifuging 1 mL of the formulations (10,000 rpm, 30 min) using an Allegra® X-15R centrifuge (Beckman Coulter, Pasadena, CA, USA). Then, 500 μL of each formulation supernatant was added to 500 μL of DMSO, and the amount of quercetin in the supernatant was quantified using an ultraviolet–visible light (UV–Vis) spectrophotometer (Jasco V-660 Spectrophotometer, Software: Spectra Manager v.2, Jasco Corporation, Easton, Maryland, USA) at 374 nm [62]. The entrapment efficiency (EE) and the drug loading (DL) were obtained using the following equations:

$$EE\;\left(\%\right)=\frac{Total\;initial\;drug-Drug\;in\;supernatant}{Total\;initial\;drug}\times 100;$$

$$DL\;\left(\%\right)=\frac{Entrapped\;drug}{Initial\;polysaccharide\;mass}\times 100$$

#### 4.3.4. Fourier-Transform Infrared Spectroscopy Evaluation

The spectra of fucoidan, chitosan, and fucoidan/chitosan nanoparticles were obtained by placing an amount of the powder of each compound in FTIR equipment (Frontier^TM^, Perkin Elmer, Santa Clara, CA, USA) equipped with an attenuated total reflectance (ATR) device and zinc selenite crystals. The samples were transferred directly into the ATR compartment, and the result was obtained by combining 16 scans. The spectra were recorded between 4000 and 600 cm^−1^ with a resolution of 4 cm^−1^.

#### 4.3.5. pH Responsiveness 

In order to evaluate the nanoparticles’ pH responsiveness, each formulation was submitted to different pH conditions, and information on the size, PDI, and zeta potential was collected. To do so, a 1 M NaOH solution or 1 M HCl solution was added dropwise to each formulation (initially at pH = 3) to obtain the different physiological pH conditions (2.5, 6.0, 7.0, and 7.4), mimicking the gastrointestinal tract environment.

#### 4.3.6. In Vitro Release Assays

The in vitro release studies were conducted according to the dialysis bag method. Briefly, 1 mL of quercetin-loaded formulation was placed in a cellulose bag (Cellu.Sept^®^T3, membrane filtration products Inc., Seguin, TX, USA), and this membrane was placed in a beaker containing 80 mL of the intended medium, and submitted to heat and stirring using a magnetic stirring plate (IKA-Werke, Staufen, Germany). To mimic human gastrointestinal conditions, biorelevant media (FaSSGF, pH 1.6 and FaSSIF, pH 6.5) were used at 37 °C under constant magnetic stirring. Then, 200 μg of free or nanoparticle-loaded quercetin was placed in the dialysis bags and transferred to 80 mL of buffer for 3 h under gastric conditions and 4 h under intestinal conditions. At regular intervals of 1 h, 50 μL of the nanoparticles was collected from the dialysis bag, before being dissolved with 500 μL of DMSO to release the quercetin, and further centrifuged to separate the polysaccharides. All samples were analyzed using a UV–Vis spectrophotometer (Jasco V-660 Spectrophotometer, Software: Spectra Manager v.2, Jasco Corporation). Mathematical models for the evaluation of drug release kinetics (zero-order, first-order, Higuchi, Peppas–Korsmeyer, and Hixson–Crowell) were fitted to the experimental data and the best-fit model was selected based on the regression coefficient (*R^2^*).

#### 4.3.7. Determination of the Antioxidant Activity Using the ABTS Assay

The ABTS radical cation decolorization test provided information about the antioxidant activity of free quercetin and quercetin-loaded nanoparticles in the range of 20 to 200 μg·mL^−1^, and was applied in a similar way to previously described methods [63,64,65]. Briefly, a mixture of equal volumes of 7 mM ABTS in water and 2.45 mM potassium persulfate aqueous stock solution was left to stand in the dark at room temperature overnight before use. To study the samples, the ABTS solution was diluted with the sample buffer to an absorbance of 0.90 ± 0.02 at 734 nm. After the addition of 100 μL of diluted ABTS solution to 100 μL of sample, the absorbance (A_Sample_) reading was taken after 15 min. In each assay, a sample buffer blank (A_Control_) was tested, and all determinations were carried out in triplicate. The percentage of radical scavenging activity (% RSC) was calculated using the following equation:

$$RCS\;\left(\%\right)=\frac{A_{Control}-A_{Sample}}{A_{Control}}\;\times \;100.$$

### 4.4. Statistical Analysis

Statistical analysis was performed using the GraphPad Prism Software (Version 6.01 for Windows; GraphPad Software Inc, San Diego, CA, USA). The *t*-test and one-way analysis of variance were used to assess the differences between formulations.

## Figures and Tables

**Table 1:** Characterization of fucoidan/chitosan (F/C) nanoparticles under distinct pH conditions of the gastrointestinal tract.

	**1F/1C**	**3F/1C**	**5F/1C**
	**Particle size (nm)**		
pH 2.5	421 ± 10	303 ± 10	355 ± 9
pH 3.0 *	427 ± 26	305 ± 10	336 ± 10
pH 6.0	999 ± 100	326 ± 9	278 ± 15
pH 7.0	2778 ± 720	297 ± 10	279 ± 10
pH 7.4	3310 ± 431	339 ± 4	388 ± 16
	**Zeta potential (mV)**		
pH 2.5	+51 ± 2	−36 ± 2	−41 ± 2
pH 3.0 *	+61 ± 2	−39 ± 3	−44 ± 3
pH 6.0	−4 ± 2	−24 ± 3	−25 ± 3
pH 7.0	−18 ± 2	−21 ± 3	−23 ± 3
pH 7.4	−14 ± 3	−23 ± 4	−23 ± 2

* Data from initial formulation.

**Table 2:** Physicochemical characterization of fucoidan/chitosan nanoparticles.

Formulation	Size (nm)	PDI	ζ Potential (mV)	EE (%)	DL (%)
1F/1C	427 ± 26	0.12 ± 0.04	+61 ± 2	-	-
Q-1F/1C	335 ± 17 ***	0.19 ± 0.01	+43 ± 3	96.8 ± 0.2	14.52 ± 0.02
3F/1C	305 ± 10	0.14 ± 0.02	−39 ± 2	-	-
Q-3F/1C	352 ± 2	0.24 ± 0.02	−29 ± 3 **	98.9 ± 0.6	14.84 ± 0.08
5F/1C	355 ± 9	0.18 ± 0.03	−44 ± 2	-	-
Q-5F/1C	356 ± 4	0.30 ± 0.02	−30 ± 3 **	99.1 ± 0.3	14.86 ± 0.05

PDI—polydispersity index; EE—entrapment efficiency; DL—drug loading; Q—quercetin. Data are represented as means ± SD (*n* = 3). Statistical differences are in relation to unloaded nanoparticles; ** p < 0.01, *** p < 0.001.

**Table 3:** Values of *R^2^* obtained from fits of different mathematical models of mechanisms of drug release to the 1F/1C, 3F/1C, and 5F/1C nanoparticles.

Mathematical Model	1F/1C	3F/1C	5F/1C
Zero-order	0.8658	0.9071	0.9600
First-order	0.7845	0.7773	0.8962
Hixson–Crowell	0.8139	0.8257	0.9221
Higuchi	0.9737	0.9762	0.9737
Korsmeyer–Peppas	0.9830	0.9790	0.9927
n-Value	0.4	0.6	0.5
